# Sotatercept: A Crosstalk Between Pathways and Activities in the Pulmonary Circulation and Blood

**DOI:** 10.3390/ijms26104851

**Published:** 2025-05-19

**Authors:** Rosalinda Madonna, Sandra Ghelardoni

**Affiliations:** 1 Department of Surgical, Medical and Molecular Pathology and Critical Area, Cardiology Division, University of Pisa, 56124 Pisa, Italy; 2 Department of Surgical, Medical and Molecular Pathology and Critical Area, Laboratory of Biochemistry, University of Pisa, 56124 Pisa, Italy; sandra.ghelardoni@unipi.it

**Keywords:** activins, bone morphogenic protein, sotatercept, pulmonary arterial hypertension, hematological disorders, anemia, bleeding

## Abstract

Sotatercept selectively binds free activins and growth differentiation factors by reproducing the binding domain of the activin receptor type IIA (ACTRIIA). The sequester of activins blunts the downstream signaling pathway, resulting in the reactivation of the bone morphogenic protein (BMP) receptor type 2 signaling and inhibition of pathological remodeling in pulmonary circulation. The balance between proliferative and antiproliferative pathways is restored, with a favorable impact on the progression of pulmonary arterial hypertension (PAH). Sotatercept, first approved for the treatment of hematological disorders such as anemia, has recently received approval as a drug in the treatment of group 1 PAH, either in United States or Europe. In this review, we will discuss the application of sotatercept and its cross reactivity in function alone or in combination with other drugs currently used for PAH. We will try also to further discuss what is known regarding the hematological effects of sotatercept, both from preclinical and clinical studies points of view, since they are the root of the side effects seen in PAH trials, such as bleeding and increased hemoglobin.

## 1. Sotatercept in Pulmonary Arterial Hypertension: Between Efficacy and Side Effects

Pulmonary arterial hypertension (PAH) is a rare disease caused by hypertrophy of the media and hyperplasia of the intima of the pulmonary arteries and arterioles, with involvement of the pulmonary interstitium [1]. This remodeling results in an increase in mean arterial pressure (mPAP) measured in the pulmonary artery during right heart catheterization (RHC) at rest and increased pulmonary vascular resistance (PVR) [1]. Pulmonary remodeling ultimately culminates in right ventricular failure and death. The blockade and eventual resolution of pulmonary remodeling are the rationale for the development of drugs that block pathological remodeling and are thus able to modify the progression of the disease. Sotatercept has recently been introduced for the treatment of PAH, as a drug with strong inhibitory activity on pathological remodeling, and therefore it is potentially able to arrest the progression of disease [2]. The STELLAR study was an international pivotal randomized clinical trial demonstrating that treatment with sotatercept of patients with group 1 PAH for 24 weeks improved 6 min walk distance (6MWD), pulmonary vascular resistance, serum levels of the N-terminal fragment of the prohormone of brain natriuretic peptide, the functional class of the World Health Organization, and the time to the first onset of non-fatal clinical worsening [2]. Since the clinical improvement noticed in the STELLAR study could not have been obtained by the current standard of care, sotatercept could represent the fourth pillar in the treatment of PAH [2,3,4,5]. In the STELLAR study, in the sotatercept arm, a higher incidence of telangiectasia and increased hemoglobin levels were, however, recorded [2]. Furthermore, the study showed an increase in bleeding events in the sotatercept group (21.5%) versus the placebo (12.5%), regardless of the decrease in platelet count [2].

Sotatercept [6] sequesters free activins that act as an inhibitory brake on bone morphogenetic protein (BMP) activity, leading to deregulated proliferation of pulmonary arteries and arterioles. BMP binds to and activates the type 2 receptor (BMPR2) that phosphorylates the transcription factor SMAD1/5/8, allowing them to translocate into the nucleus and bind to specific sites on DNA. This binding inhibits DNA replication and the cell cycle in endothelial cells and smooth muscle cells [6]. Activins are a homodimeric polypeptide growth factor highly homologous to TGF-β that interact with the Alk1 receptor [6]. Alk1 is a transmembrane protein with serine/threonine kinase activity that forms a heteromeric complex and phosphorylates the nuclear transcription factor Suppressor of Mothers against Decapentaplegic (SMAD) homolog 2 and homolog 3 (SMAD2/3). Activin–Alk1 binding induces DNA replication and cell cycle activity in endothelial cells and smooth muscle cells, leading to a variety of pulmonary conditions, such as interstitial pulmonary fibrosis and idiopathic or hereditary PAH [6].

Sotatercept is administered subcutaneously and involves titration from a baseline dose of 0.3 mg/kg to 0.7 mg/kg once every 3 weeks. If we analyze the curve of the STELLAR study in which the effects on time to clinical worsening of the arm treated with background therapy and the arm treated with sotatercept on top of background therapy are shown, we note how the curves diverge significantly and very early [2]. An effect that suggests that sotatercept not only has an inhibitory action on pathological remodeling per se (which requires longer times to manifest itself) but evidently also has off-target effects on other signaling pathways that lead to vasodilation, is an effect that appears much earlier than the anti-remodeling effect alone. There are several points of interaction between the sotatercept signaling pathway and other signaling pathways on which other vasodilatory drugs specific for PAH act. First, Alk1 co-localizes with endothelial nitric oxide synthase (eNOS) in endothelial caveolae, where both interact with caveolin-1 [7]. Treatment of pulmonary endothelial cells with activin A reduces eNOS expression [8]. Furthermore, ET-1 increases Alk expression via the Gi/RhoA/Rho kinase pathway. Activation of Gi and RhoA is associated with Alk promoter activity via Sp-1 and Alk mRNA stability [9]. In light of the extensive interactions between NO, ET-1, and activin, it is possible that sotatercept not only reduces the dysregulated proliferation of pulmonary circulation vessels but also regulates their vasodilation through sequestration of activin, thereby removing the inhibitory brake on eNOS.

In addition to its effects on pulmonary circulation, sotatercept has several hematologic effects that may suggest its use in the treatment of certain blood disorders [10], but they also may explain the side effects of the drug in the PAH trials.

In this review, we will discuss the application of sotatercept, with the aim of analyzing, in depth, its cross reactivity in function and signaling alone or in combination with other drugs currently used in PAH. We will try also to further understand the hematological effects of sotatercept, which are the root of the side effects seen in PAH trials, such as bleeding and increased hemoglobin.

## 2. Crosstalk Between the Activin System and Other Signaling

### 2.1. The Activin System

The activin system is a signal transduction system widely distributed in tissues, which controls several mechanisms in body development, from embryogenesis to adulthood [11]. Activins belong to the transforming growth factor (TGF)-β superfamily and bind to a combination of receptors with serine/threonine kinase activity. The binding to activin receptor-like type 1 (ALK1) stimulates the kinase activity of the intracellular domain of the receptor, inducing the phosphorylation of transcription factor Suppressor of Mothers against Decapentaplegic (SMAD) homolog 2 and homolog 3 and the subsequent formation of the phosphoSMAD2/3/4 complex, which translocates to the nucleus [12]. The SMAD complex can cooperate with a plethora of transcription factors targeting various genes (Miyazawa K, Itoh Y, Fu H, Miyazono K. Receptor-activated transcription factors and beyond: multiple modes of Smad2/3-dependent transmission of TGF-β signaling [11]. and promoting different and opposing cell responses, among them plasminogen activator inhibitor-1 (PAI-1), elevated in arterial plaques; vessel fibrosis; arteriosclerosis; and thrombosis, inhibitor of fibrinolysis [10,11], as well as proteins promoting epithelial–mesenchymal transition such as occludin, E-cadherin, or snail; proteins inducing cell growth and survival in cancer such as the GTP-binding protein RAD; and a protein inhibiting cell growth c-Myc, p21.

Several ligands can bind and activate the activin system, among them activin A, growth differentiation factor (GDF)11, and bone morphogenetic proteins (BMP)10 promoting the expression of genes that regulate several cellular processes including cell cycle, proliferation, differentiation, extracellular matrix (ECM) formation, erythropoiesis, and apoptosis [13]. An excessive stimulation of activin signaling by activin A or a lack of inhibition exerted by the BMP system evokes an imbalance between activin and BMP signaling and produces pro-fibrotic effects or alteration in erythropoiesis, which underly several pathological conditions such asthma, cardiovascular diseases, cancer, inflammation, interstitial pulmonary fibrosis, and PAH [10,14,15], and BPM10, considered a biomarker in heart diseases, including atrial fibrillation and heart failure [16,17], is involved in the physiological function of the vascular endothelium by modulating remodeling and angiogenesis [18]. BMP10 forms heterodimers with BMP9 and subsequently a receptor complex involving ALK1, bone morphogenic protein receptor 2 (BMPR2), and endoglin, allowing the maintenance of the balance between angiogenesis and maturation of lymphatic vessels by regulating the differentiation of the vascular endothelium in endothelial cells and morphogenesis [19]. The dysregulation of the complex due to mutations can alter the integrity and functionality of the pulmonary vascular endothelium leading to PAH [18].

**Figure 1:**
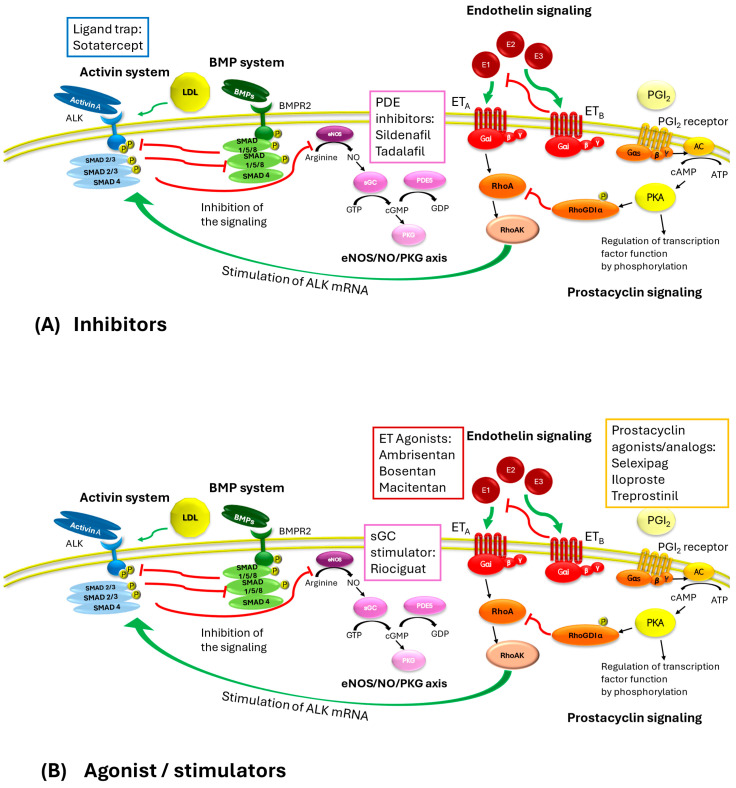
Summary of the signaling involved in pathophysiology of PAH and targeted by drugs used in the treatment of PAH. (**A**) Ligand trap and inhibitors of phosphodiesterase 5; (**B**) agonists or stimulators. Black arrows indicate downstream signal; red arrows indicate inhibition of the signaling; green arrows indicate stimulation of the signaling. ALK: activin A receptor; SMAD: Suppressor of Mothers against Decapentaplegic; BMP: bone morphogenic protein; BMPR2: bone morphogenic protein receptor 2; E1,2 3: endothelin 1,2,3; ETA: endothelin receptor type A; ETB: endothelin receptor type B; eNOS: endothelial nitric oxide synthetase; LDL: low-density lipoprotein; sGC: soluble gluanylyl cyclase; PDE5: phosphodiesterase 5; PKG: protein kinase G; PKA: protein kinase A; Gαiβγ: G protein, subunits αi,β,γ; Gαsβγ: G protein, subunits αs,β,γ; AC: adenylyl cyclase; PGI2: prostacyclin; RhoAK: RhoA kinase; RhoGDIα: Rho guanine nucleotide dissociation inhibitor α.

The activins represent a critical signaling pathway also in hematological disorders. Overstimulation by GDF11 has impacts at different stages of erythropoiesis from the differentiation of erythroid burst-forming units to the proliferation of erythroid progenitor cells [20]. Moreover, its overexpression, due to oxidative stress or alpha-globin precipitation, can evoke an autocrine amplification loop [21] and an excessive number of immature erythroblasts blocking terminal erythroid maturation [21]. Loss-of-function mutations in SEC23B and high GDF11 levels have also been observed [22]. These effects lead overall to several blood disorders including beta-thalassemia, myelodysplastic syndromes, dyserythropoietic anemias, and multiple myeloma, which are counteracted by treatments with sotatercept [10]. Moreover, as already indicated, activin signaling co-localizes and interacts with other pathways that are described in the following paragraphs (Figure 1).

### 2.2. The Bone Morphogenetic Protein System

As activins, bone morphogenetic proteins (BMPs) are members of the transforming growth factor-β (TGF-β) superfamily. BMP signaling exerts antifibrotic effects and is widely distributed in tissues and organs, including the lungs, maintaining the integrity of the endothelial wall of arteries [15,23]. BMP receptors are serine/threonine kinases, and binding to the type 2 receptor (BMPRII) triggers the activation by phosphorylation of the type 1 receptor. The complex targets the receptor-regulated SMADs by phosphorylation. The SMADS mainly involved are SMAD1, SMAD5, and SMAD9 [11], which aggregate upon phosphorylation with SMAD4 and translocate to the nucleus, regulating the expression of genes such as inhibitor DNA binding 1, which may play a critical role in cell growth, senescence, and differentiation, and inhibitor of DNA binding 2 that negatively regulates cell differentiation and the cell cycle in endothelial and smooth muscle cells [15,23]. Dysregulation in BMP signaling underlies inherited and non-hereditary forms of PAH [24,25], leading to a decrease in antiproliferative effects. Several genes encoding proteins involved in the BMP signal transduction and highly expressed in vascular endothelial cells are mutated in PAH, including BMPR2, ALK1, and endoglin (ENG) [23], leading to endothelial dysfunction, i.e., cell apoptosis, compromised barrier function, altered vasoactive mediator release, and permeability in lung vasculature [23]. Mutations in ALK1 and ENG are associated with different types of hemorrhagic telangiectasia, also known as Rendu–Osler–Weber syndrome [26]. Dysregulated endothelial BMP signaling is no longer able to attenuate the activin system, provoking an imbalance that triggers pulmonary vascular remodeling (Figure 1).

### 2.3. Endothelin Receptor Signaling

Sugimoto et al. demonstrated that endothelin-1 and the activin systems can interact with each other through the Gi/RhoA/Rho kinase pathway in human pulmonary artery endothelial cells [8]. The crosstalk between the two pathways potentiates the vasoconstrictor action of the ET-1 signaling [9] whose activation upregulates the activin receptor-like kinase 1 expression. Endothelin receptor antagonists such as ambrisentan, bosentan, and macitentan are oral drugs currently approved for the treatment of PAH [27] that prevent binding between the endothelin receptor and its natural ligands [27]. Bosentan and macitentan are non-peptide dual antagonists of the binding of endothelin-1 to ET A and ET B receptors in human pulmonary arterial smooth muscle cells [28,29], while ambrisentan is a selective potent antagonist against the ET_A_ receptor [30]. The endothelin receptor signaling consists of two seven-transmembrane (7TM) receptor subtypes, endothelin receptor type A (ET_A_), and endothelin receptor type B (ET_B_), which, binding endothelins, can activate multiple types of G proteins [31]. They modulate several physiological processes including vasoconstriction, vasodilation, growth, survival, invasion, and angiogenesis [31]. Endothelins are a family of 21 amino acid peptides produced by the endothelium: endothelin 1 (ET-1) and ET-2 activate both receptor subtypes with equal affinity, whereas ET-3 has a lower affinity for ET_A_ [32]. Among all endothelins, the first-discovered peptide endothelin-1 presents the strongest vasoconstrictor effect by interacting with the ET_A_ receptor [31]. Interestingly, the binding with ET_B_ mediates vasodilation and the clearance of circulating endothelin-1 by lysosomal degradation [31], promoting the development of novel pharmacological strategies in the form of agonists of ET_B_ agonists [31] (Figure 1).

### 2.4. CGMP/Phosphodiesterase Signaling

The activin system can downregulate endothelial nitric oxide synthetase (eNOS), disrupting the NO signaling and thus leading to an increase to pulmonary pressure [7,8]. The NO-soluble guanylate cyclase–cyclic guanosine monophosphate (NO-sGC-cGMP) axis is one of the types of signaling that enables vasodilation; regulates blood pressure; improves vascular function; and inhibits inflammation, fibrosis, and cell proliferation [33]. Phosphodiesterase 5 catalyzes the degradation of the second messenger cGMP, and inhibitors of this enzyme are effective treatments in attenuating pulmonary hypertension and vascular remodeling in PAH [34]. PDE5 inhibitors and soluble guanylate cyclase stimulators, including sildenafil, tadalafil, and riociguat can produce vasodilation or attenuate PAH remodeling by maintaining high concentrations of intracellular cGMP, which induce antiproliferative, antifibrotic, and anti-inflammatory effects [9,33] (Figure 1).

### 2.5. Prostacyclin System

In 2024, Savale et al. [34] observed a downregulation of the insulin-like growth factor binding protein (IGFBP) 7 in patients treated with sotatercept compared to the placebo; IGFBP7 can stimulate prostacyclin production and cell adhesion, but it is also a biomarker for PAH whose increased expression may be associated to fibrotic changes in pulmonary vasculature [35,36]. Prostacyclin (PGI2) is a potent vasodilator with antiplatelet and antiproliferative properties that can protect against atrial fibrosis [37]. PGI_2_ is primarily synthesized in vascular endothelial cells, vascular smooth muscle cells, and fibroblasts from arachidonic acid by the sequential catalysis of cyclooxygenase-2 and PGI synthase upon endogenous or exogenous stimuli [38]. When it binds its receptors, a Gs-type G protein-coupled receptor, PGI2, evokes the activation of adenylyl cyclase and thus the synthesis of cyclic adenosine 3′,5′-monophosphate (cAMP), which in turn binds and activates protein kinase A, inducing the final effects [38]. PGI_2_ acts as potent anti-fibrotic and vasodilator agent by inhibiting cell proliferation; smooth muscle contraction; platelet aggregation, decreasing the synthesis of proteins of the extracellular matrix (ECM); and cardiomyocyte hypertrophy [38,39]. The prostacyclin pathway promotes the inhibition of fibrosis by PKA, which phosphorylates Rho guanine nucleotide dissociation inhibitor α (RhoGDIα), favoring its association with RhoA-GTP, which results in RhoA inhibition, promotor of fibrosis. Furthermore, PGI2 signaling evokes the dissociation of raptor protein from the mechanistic target of rapamycin complex (mTORC) 1 and induces cAMP-response element binding protein (CREB) phosphorylation inhibiting muscle-cell proliferation and migration, vasoconstriction, and extracellular matrix remodeling, and it exerts an anti-apoptotic role through peroxisome proliferator-activated receptor (PPAR)β in endothelial cells and PPARα in vascular smooth muscle cells. Treatment with prostacyclin analogs such as selexipag, iloprost, treprostinil, or epoprostenol can improve clinical worsening in PAH patients over background targeted therapies, by improving exercise capacity, mean pulmonary artery pressure, and cardiac index, even though an overall risk of adverse events cannot be excluded [40] (Figure 1).

## 3. Sotatercept Beyond the Anti-Remodeling Effect: Impact on Cell Metabolism and Regulation

**Figure 2:**
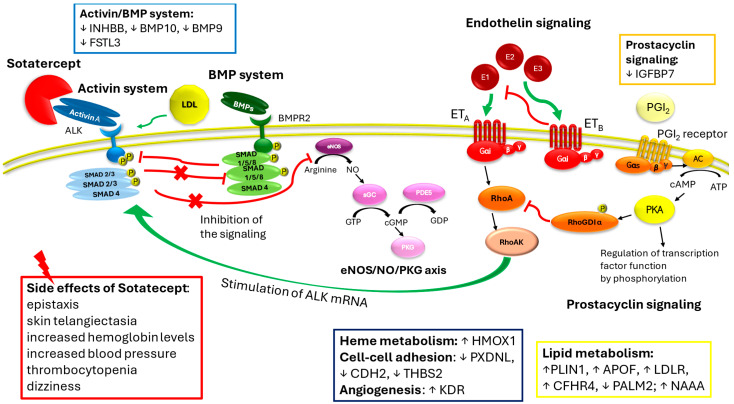
Sotatercept beyond the activin system: summary of the biomarkers potentially altered by sotatercept and their impact on PAH pathophysiology signaling and cell metabolism. Markers altered by administration of sotatercept were grouped depending on the metabolism or signaling system to which they belong; ↓: downregulation of the protein expression; ↑: upregulation of the protein expression. Black arrows indicate downstream signal, red arrows indicate inhibition of the signaling, and green arrows indicate stimulation of the signaling. INHBB, inhibin subunit beta B; IGFBP7, insulin-like growth factor binding protein 7; FSTL3, follistatin-like 3; PLIN1, perilipin1; APOF, apoprotein F; LDLR, LDL receptor; CFHR4, complement factor H-related 4; PALM2, paralemmin; NAAA, N-acylethanolamine acid amidase; HMOX1, inducible heme oxygenase-1; PXDNL, peroxidasin-like; CDH2, cadherin 2 or N-cadherin; THBS2, thrombospondin 2; ALK: activin A receptor; SMAD: Suppressor of Mothers against Decapentaplegic; BMP: bone morphogenic protein; BMP9/10, bone morphogenic protein 9/10; BMPR2, bone morphogenic protein receptor 2; E1,2,3, endothelin 1,2,3; ETA, endothelin receptor type A; ETB, endothelin receptor type B; eNOS, endothelial nitric oxide synthetase; sGC, soluble gluanylyl cyclase; PDE5, phosphodiesterase 5; PKG, protein kinase G; PKA, protein kinase A; Gαiβγ, G protein, subunits αi,β,γ; Gαsβγ, G protein, subunits αs,β,γ; AC, adenylyl cyclase; PGI2, prostacyclin; RhoAK, RhoA kinase.

Complementary experimental and genetic models of PAH reveal therapeutic anti-inflammatory activities of the activin receptor type IIA-fragment crystallizable (ActRIIA-Fc) fusion protein that, together with its known anti-proliferative effects on vascular cell types, could underline the clinical activity of sotatercept as either monotherapy or an add-on to current PAH therapies [41]. Sotatercept was synthetized to recruit and trap activin A or similar ligands, inducing a reduction of activin signaling. However, it should be regarded as relevant that Savale et al. (2024), in their proteomic study analyzing blood of PAH patients treated with sotatercept for 24 weeks vs. the placebo, highlighted that not only the activin system was affected but also the expression of proteins that have key roles in inflammation, in cardiovascular and oxidative stress systems, and, last but not least, in cellular metabolism, often dysregulated in PAH patients [34]. Among them, the authors found a downregulation in inhibin subunit beta B (INHBB) expression, a preproprotein that is proteolytically cleaved to generate a subunit of the dimeric activin and inhibin protein complexes. Sotatercept treatment alters also markers involved in recruitment and activation of inflammatory cells and in oxidative stress. Among those in recruitment, there is also the insulin-like growth factor binding protein 7 (IGFBP7), which binds IGF-I and IGF-II with relatively low affinity and stimulates prostacyclin production and cell adhesion. Sotatercept also affects proteins implicated in lipid metabolism. Perilipin1 (PLIN1), apoprotein F (APOF), LDL receptor (LDLR), complement factor H related 4 (CFHR4), paralemmin (PALM2), and N-acylethanolamine acid amidase (NAAA) are all proteins involved in the metabolism of complex lipids or lipid transport through lipoproteins. Interestingly, except for PALM2, they are all upregulated. Low-density lipoproteins (LDLs) can bind to activin A receptor-like kinase 1 with lower affinity than LDL receptors on the cell surface, promoting transcytosis in endothelial cells [42,43]. The binding is saturated only at hypercholesterolemic concentrations and avoids lysosomal degradation [42]. Interestingly, according to Savale et al. [34], in PAH patients treated with sotatercept the LDL receptor is upregulated compared to the placebo, indicating a compensatory mechanism due to lack of activin A. Furthermore, sotatercept, preventing activation of the Smad2/3 pathway by trapping activins or GDF11, reverses the process that leads to hematological disorders related to abnormal activin signaling. Blunting the activin system induces a blocking of erythroid progenitor cell differentiation and of excessive numbers of immature erythroblasts and re-establishes the erythroid progenitor cell development or osteoblast differentiation, leading to an overall increase in hemoglobin and hematocrit [10] (Figure 2).

## 4. Hematological and Vascular Effects of Sotatercept

The most common adverse events that occurred with sotatercept compared to the placebo include epistaxis, dizziness, skin telangiectasia, increased hemoglobin levels, thrombocytopenia, and increased blood pressure [44,45,46]. In STELLAR, one instance of gastrointestinal bleeding was noted in the treatment group. Overall, bleeding events were higher in the sotatercept group compared with the placebo, although they were not associated with a decrease in platelet numbers. Treatment with sotatercept increases the expression of the inducible heme oxygenase-1 (HMOX1) [34] This enzyme belongs to the heme oxygenase family and catabolizes the heme group to form biliverdin, which is further metabolized to bilirubin and carbon monoxide by biliverdin reductase. The role of overexpression of HMOX1 is still controversial and contrasting results are reported. In experimental models of intracerebral hemorrhage, its overexpression induces a proinflammatory response in microglia and disrupts the balance of iron metabolism [47,48], while in astrocytes or in a subarachnoid hemorrhage mouse model HMOX-1 overexpression provides neuroprotective effects against intracerebral hemorrhage [49,50] or against Dengue virus-induced vascular endothelial dysfunction and leakage [51]. Interestingly, sotatercept reduces the expression of follistatin-like 3 (FSTL3) [35], which is a strong inhibitor of the activin system but exerts a weak inhibitory effect on BMP signaling [52]. Furthermore, FSTL3 cooperates with integrins in regulating the adhesion of hematopoietic cells to fibronectin [52] and seems to promote vascular endothelial cells from induced pluripotent stem cells by upregulating endothelin-1 [53]. Accordingly, BMP9 and BMP10 are downregulated in sotatercept treatment [35], worsening the pathological spectrum toward vascular lesions, abnormal blood vessels, and hereditary hemorrhagic telangiectasia, in which vascular endothelial cells show a hyperactivation of the VEGFR2 pathways [35]. Consistent with these findings, Savale et al. (2024) also found an increase in expression of KDR (kinase insert domain receptor), which is a type III receptor tyrosine kinase, one of the two receptors for the VEGF [34]. Peroxidasin-like (PXDNL) is another marker downregulated by sotatercept [35]; it belongs to the peroxidase gene family and is highly expressed in the cardiovascular system. Early studies indicate that PXDNL is involved in the extracellular matrix formation with the potential of antagonizing PXDN activity [54]. Lastly, other proteins such as cadherin 2 (CDH2, N-cadherin) and thrombospondin 2 (THBS2), which are involved in cell–cell adhesion and in maintaining tissue integrity and cell proliferation, are also downregulated in presence of sotatercept [35]. Given the above, alteration in expression of these markers may contribute to the adverse effects encountered during the treatment with sotatercept (Figure 2). These findings reveal that the whole mechanism of sotatercept is still not uncovered, as also it is unknown whether sotatercept impacts marker expression directly or if the protein modulation is a consequence of the activin-signaling inhibition.

## 5. Effects of RAP-011, the Murine Orthologue of Sotatercept, in Experimental Models of Hematologic Disorders

Preclinical evaluation of sotatercept has been widely performed through in vivo and in vitro experimental models of blood diseases and pulmonary hypertension with the aim of better understanding the crosstalk between BMPs and activin signaling, which are often compromised in anemia [10]. Erythropoiesis is a process that can be affected by imbalance between activin and the BMP signaling pathway, leading to a reduction in red blood cell production and, thereby, in is found in various forms of anemia. Since sotatercept is a chimeric protein containing the extracellular domain of the activin receptor 2A (ACVR2A) fused to the Fc domain of human IgG1, in preclinical studies its murinized counterpart, RAP-011, which is composed of the soluble extracellular domain of ActRIIA fused to a murine IgG2a-Fc [55], has been widely assessed. In 2016, authors observed that the infusion of RAP-011 in mice produced a rapid increase in hematocrit, hemoglobin levels, and in red blood cell count; these effects were attributed to the ability of RAP011 to revert the inhibition induced by the activin system on late-stage erythroid precursors in the bone marrow and to enhance erythropoietin and erythroid burst-forming units [56]. Interestingly, RAP-011 required bone marrow accessory cells to rescue inhibition evoked by activin signaling and, thereby, to restore physiological erythropoiesis [56]. To better understand the mechanism underlying the blood disorder and the efficacy of RAP-011 in vivo, several models of anemia were produced. Ear et al., in 2015, produced a zebrafish model harboring a mutation mimicking the dysfunction of 5q-syndrome, a form of myelodysplastic syndrome [57]. In a model representative of Diamond Blackfan anemia, in the RPL11 ribosome-deficient zebrafish, administration of RAP-011 produced a remarkable increase in hemoglobin concentration by stimulating erythropoiesis through the sequester of lefty1, likely being implicated as a signaling marker in erythroid cell development [57]. Recently, RAP-011 was also found effective in a model of congenital dyserythropoietic anemia type II, an anemia characterized by ineffective erythropoiesis due to maturation arrest of erythroid precursors [58]. The study demonstrated that the administration of RAP-011 could increase hemoglobin concentration in transgenic and wild-type models without inducing splenic depletion of iron store, as differently observed upon infusion of erythropoietin, indicating that sotatercept may have a role in in the management of iron overload in patients with congenital dyserythropoietic anemia type II. In transgenic mice, the mechanism involved a restoring of gene expression of erythroid markers by inhibiting of the phospoSMAD2 pathway [58]. RAP-011 was also evaluated in a mouse model of β-thalassemia intermedia [21]. As reported, treatment with RAP-011 ameliorated ineffective erythropoiesis, corrected anemia, and limited iron overload, as already observed in other animal models, by acting as a binding trap for GDF11. The inhibition of the overstimulation of GDF11 signaling improved β-thalassemia effects by reducing the abnormal production of reactive oxygen species and the precipitation of α-globin membranes and by favoring a balance between immature/mature erythroblasts via apoptosis [21]. Several studies performed in in vitro models have confirmed in vivo findings and improved the comprehension of the underlying mechanisms. In 2012, Iancu-Rubin et al. [59] observed that ACE-011 (sotatercept) did not directly affect erythroid differentiation of human CD34(+) cells but could improve erythropoiesis inhibited by conditioned media produced by bone marrow stromal cells, indicating that ACE-011 might influence the production of inhibitory factors from bone marrow stromal cells. It is interesting to point out that, since the activin signaling system is also strictly connected to the bone morphogenesis, preclinical studies have been addressed to evaluate treatments with RAP-011 even in experimental models of mineral and bone disorders, which often are associated to chronic kidney and cardiovascular disease [60,61]. In early studies in female cynomolgus monkeys (Macaca fascicularis), treatments with ACE-011 could produce an improvement of bone strength and matrix mineralization by rebalancing bone resorption and formation, which are impaired by overstimulation of the activin pathway [62]. A rat model of closed-fracture RAP-011 was able to promote bone formation during repair but not to optimize callus bone quality [63].

In conclusion, preclinical evidence evaluated in vivo and in vitro experimental models indicates that sotatercept can restore functional erythropoiesis and bone formation by inhibiting activin signaling, indicating it could be a new drug strategy.

## 6. Sotatercept in Hematological Clinical Studies: A Summary of Findings

According to preclinical studies, sotatercept and its murinized analogue exhibit the ability to restore erythropoiesis and bone formation. Similar findings have been observed in clinical trials. In the first clinical trial on healthy postmenopausal women, sotatercept produced a significant and persistent improvement in reverting erythropoiesis and bone mineral density [64]. In a phase IIa trail, sotatercept was administered at different concentrations (0.1–0.5 mg/kg vs. placebo) to patients affected by multiple myeloma for four-week cycles, in combination with oral therapy of melphalan, prednisolone, and thalidomide. Sotatercept enhanced hemoglobin levels compared to the baseline as well as the duration of the increase vs. the placebo. Moreover, anabolic improvements in bone mineral density and in bone formation were recorded. Multiple doses of sotatercept with the combined treatment appeared safe and generally well tolerated by patients [65]. In phase II studies, administration of sotatercept in patients with metastatic breast cancer or with solid tumors treated with platinum-based chemotherapy induced an increase in hemoglobin of ≥1.0 g/dL [66]. In these clinical trials, sotatercept proved to be effective with a safety profile in the treatment of anemia, which was evoked by cancer therapies. In a prospective, open-label, single-institution, investigator-initiated phase II clinical study, 56 anemic patients with primary myelofibrosis or post polycythemia vera/essential thrombocythemia myelofibrosis were enrolled [67]. The study also included patients who were already in ruxolitinib therapy. The primary endpoint of the study was achieved by 30% of patients in 84 days either in monotherapy with sotatercept or combination therapy with ruxolitinib, with a duration of response ranging up to 9 years (approximately 23 and 20 months in monotherapy and combination therapy, respectively). Participants in combination therapy experienced more serious adverse effects (48%) then patients in monotherapy (31%). The most frequent (>5%) serious adverse events were anemia, fever, infection, and neoplasms, while the most common minor adverse events were gastrointestinal disorders, pain, bleeding, and flu-like symptoms. The open-label, dose-finding, multicenter phase II study (NCT01571635) randomized 16 adults with transfusion-dependent β-thalassemia and 30 adults with non-transfusion-dependent β-thalassemia to sotatercept versus the placebo for two years. The study met its primary and secondary outcomes, observing a significant reduction in transfusion burden in patients with transfusion-dependent β-thalassemia, and an increase in hemoglobin levels from the baseline in patients with non-transfusion-dependent β-thalassemia over 12 weeks. Sotatercept was overall effective and well tolerated since all patients in the study experienced non-serious adverse effects, mainly abdominal pain and other gastrointestinal disorders [67]. A multicenter, open-label, dose-funding phase II clinical study (NCT01736683) enrolled 74 adult patients with anemia and low or intermediate-1 risk of myelodysplastic syndromes or non-proliferative chronic myelomonocytic leukemia (CMML) and who were ineligible for or refractory to erythropoiesis-stimulating agents (ESAs). Sotatercept was randomly administered at doses of 0.1 and 0.3 mg/kg and non-randomly at 0.5, 1, and 2 mg/kg doses. The primary endpoint was to determine the dose that achieved hematological erythroid improvement (HI-E) prior to completion of five treatment cycles, defined as an increase of >1.5 g/dL hemoglobin or a decrease of >4 units of transfused RBCs, both maintained for 56 days over a period of 8 weeks. Sotatercept (0.3 mg/kg) was effective in improving anemia and in reducing RBC transfusion in patients with lower-risk myelodysplastic syndromes [67].

## 7. Conclusions and Future Perspectives

Sotatercept, in sequential combination with background therapy and for a duration of 24 weeks of treatment, has demonstrated an unequivocal improvement in several functional and hemodynamic endpoints in patients with PAH group 1 (idiopathic, heritable, drug- or toxin-induced, associated with connective tissue disease or associated with corrected congenital shunts). In particular, sotatercept improved the hard endpoint of time to clinical worsening.

Despite this, attention must be paid to the side effects of the drug, whose actions range from pulmonary circulation to the vessels, to cellular metabolism, and to the blood.

Although the results indicate that the administration of sotatercept is well tolerated in anemic patients, there are no data on the effects in non-anemic patients and in long-term follow-up. In particular, it is not known what the effect of the increase in hemoglobin concentration and red blood cell counts may be in non-anemic patients with PAH. Likewise, the incidence of bleeding in patients treated with sotatercept and the possible long-term clinical implications are unknown. Side effects are part of the bunch of drug effects and new experimentations that are required to define the balance between efficacy and adverse effects. Further studies with higher sample sizes are needed to better define the safety of the drug and its efficacy, especially in the long term, both in the hematological field and in PAH. Only pharmacovigilance can reveal the real efficacy of sotatercept, after a large cohort of PAH or anemic patients have experienced sotatercept treatment.

## Data Availability

No new data were created or analyzed in this study. Data sharing is not applicable to this article.
